# Exploring polymorphisms in genes encoding growth factors associated with non-syndromic cleft lip with or without cleft palate and tooth agenesis

**DOI:** 10.1590/1678-7757-2024-0501

**Published:** 2025-03-14

**Authors:** Gabriela FONSECA-SOUZA, Vitória Somma TESSARI, Rafaela SCARIOT, Christian KIRSCHNECK, Ricardo Della COLETTA, Erika Calvano KÜCHLER, Juliana FELTRIN-SOUZA

**Affiliations:** 1 Universidade Federal do Paraná Setor de Ciências da Saúde Departamento de Estomatologia Curitiba Paraná Brasil Universidade Federal do Paraná, Setor de Ciências da Saúde, Departamento de Estomatologia, Curitiba, Paraná, Brasil.; 2 University of Bonn Department of Orthodontics Bonn Germany University of Bonn, Department of Orthodontics, Bonn, Germany.; 3 Universidade Estadual de Campinas Faculdade de Odontologia de Piracicaba Departamento de Diagnóstico Oral Piracicaba São Paulo Brasil Universidade Estadual de Campinas, Faculdade de Odontologia de Piracicaba, Departamento de Diagnóstico Oral e Programa de Pós-graduação em Biologia Buco-Dental, Piracicaba, São Paulo, Brasil.; 4 University of Bonn Department of Orthodontics Bonn Germany University of Bonn, Department of Orthodontics, Bonn, Germany.

**Keywords:** Cleft lip, Cleft palate, EGF family of proteins, Transforming growth factors, Anodontia

## Abstract

**Objective:**

To evaluate the association between non-syndromic cleft lip with or without cleft palate (NSCL±P) and tooth agenesis (TA), as well as the association of both conditions with polymorphisms in genes encoding growth factors.

**Methodology:**

This cross-sectional study included children with NSCL±P and a control group of children without NSCL±P. Permanent teeth TA (excluding third molars) was evaluated using panoramic radiographs by a trained examiner. Only TA located outside the cleft was considered in the NSCL±P group. Genetic polymorphisms in Transforming Growth Factor Beta 1 (*TGFB1*)–rs1800470 and rs4803455–Transforming Growth Factor Beta Receptor 2 (*TGFBR2*)–rs3087465 and rs764522–Epidermal Growth Factor (*EGF*)–rs4444903 and rs2237051–and Epidermal Growth Factor Receptor (*EGFR*)–rs2227983– were genotyped by real-time PCR allele discrimination from buccal cell samples. Associations were tested by uni and multivariable Poisson regression models (5% significance level).

**Results:**

A total of 243 children–127 with NSCL±P (mean age = 8.80±2.14 years) and 116 without NSCL±P (mean age = 8.58±2.03 years) were included. TA was more frequent in the NSCL±P group (23.8%) than in the control group (6.2%) (p<0.01). The *EGF* rs2237051 was significantly associated with NSCL±P, independently of the other variables (PRa=1.41; p=0.042). Regarding TA, only the cleft presence was associated with a higher prevalence of TA regardless of different variables (PRa=3.70; p=0.001). There was no association between TA and the investigated genetic polymorphisms. When TA and NSCL±P were considered together, a borderline association was observed with rs1800470 in *TGFB1* (p=0.06).

**Conclusion:**

NSCL±P is associated with TA outside the cleft area. The *EGF* rs2237051 was associated with NSCL±P. Polymorphisms in genes encoding growth factors are not associated with TA.

## Introduction

The development of teeth and facial structures, including the lip and palate, present a close relationship considering timing and anatomical position during human embryogenesis.^1^ The primary lip, responsible for the formation of the upper lip and alveolar process of maxillary incisors, is formed by the fusion of the medial nasal and maxillary processes between the fourth and sixth weeks of embryological development. Subsequently, the secondary palate is formed between the seventh and twelfth weeks by the fusion of the palatine process of maxilla.^1,2^ Whereas primary teeth begin development at approximately six weeks, and permanent teeth begin at approximately twenty weeks.^3^ Disruptions in these processes may result in non-syndromic cleft lip with or without cleft palate (NSCL±P) and tooth abnormalities, including tooth agenesis (TA), supernumerary teeth, developmental enamel defects, microdontia and taurodontism.^4^

NSCL±P and TA are two of the most reported craniofacial developmental congenital anomalies in humans.^5^ A previous systematic review^4^ observed that individuals with oral clefts have almost 20 times more chances of having TA than non-affected individuals. The reported prevalence ranges from 13.1% to 77.3%.^4^ Patients with NSCL±P may present TA both inside and outside the cleft area.^6,7^ Inside the cleft area, the most common missing tooth is the lateral incisor.^7,8^ In this region, the cleft defect itself and surgical traumas may disturb the mesenchymal tissue and blood supply needed for dental development.^9^ Outside the cleft region, any teeth can be missing, however, the absence of second premolars is more commonly observed.^10 11^ TA away from the cleft area may suggest that these craniofacial abnormalities share a similar genetic background.^5^

Evidence from animal models and genetic studies in humans have been demonstrating the critical role of growth factors, like transforming growth factor-beta 1 (TGFB1) and epidermal growth factor (EGF) in craniofacial development, including palate closure and proper dental development.^12-19^ TGFB1 is part of a superfamily of secreted proteins that bind with transforming growth factor B receptor 2 (TGFBR2),^20^ playing a role in cell proliferation, differentiation, and growth in several tissues, including bone, epithelial and connective.^15^ This protein and its receptor are reported to have a function during palate development, acting in cell proliferation, growth, and fusion of the palatal shelves.^14,18,21^During all stages of odontogenesis, TGFB1 also regulates cell proliferation, being expressed in both the mesenchyme and epithelia.^19^

EGF is a mitogenic factor that stimulates cell proliferation by binding to its receptor (Epidermal Growth Factor Receptor–EGFR). Both EGF and EGFR encoding genes are reported to be expressed during palatogenesis.^13^ Previous animal model studies demonstrate that alterations in the expression of these proteins are associated with failures in the fusion of palate shelves.^12,17^ Regarding dental development, a study observed that the EGF is expressed in the developing mandible before the appearance of dental lamina, affecting its pattern.^16^ Besides that, EGF seems to prevent odontoblast and ameloblast cell differentiation.^22^

Few studies investigated the association of polymorphisms in genes encoding growth factors with human craniofacial phenotypes. Gerber, et al.^23^ (2021) found an association between variations in tooth size and rs1800470 in *TGFB1* and rs4444903 in *EGF*. Kirschneck, et al.^24^ (2022) found that the rs3087465 in the promoter region of the *TGFBR2* is associated with mandibular retrognathism. Therefore, this study aimed to evaluate the association between TA and NSCL±P, and if these craniofacial alterations are associated with genetic polymorphisms in *TGFB1* (rs4803455 and rs1800470), *TGFBR2* (rs764522 and rs3087465), *EGF* (rs4444903 and rs2237051), and *EGFR* (rs2227983).

## Methodology

This cross-sectional study with a comparison group is reported according to the STREGA checklist–Strengthening the Reporting of Genetic Association Study Statement Checklist.^25^ This study follows the Declaration of Helsinki and was approved by the Research Ethics Committee of the State Health Department of Paraná (protocol number 5.100.185), and by the Human Research Ethics Committee of the Health Sciences of the Federal University of Paraná (protocol number 3.752.172), both located in Curitiba, Paraná, Brazil. The assent and consent were obtained from the children and their legal guardians, respectively.

### Sample screening

The sample included children aged between 6 and 14 years old of both sexes, without history of extractions of permanent teeth. Two groups of children were evaluated: a group of children with NSCL±P (NSCL±P group) and a control group of children without NSCL±P (control group). The group with NSCL±P was recruited from a reference center for craniofacial anomalies treatment (Centro de Atendimento Integral ao Fissurado Labiopalatal –CAIF–Curitiba, Paraná, Brazil). CAIF was the first integrated center for the treatment of craniofacial anomalies in southern Brazil. This center receives patients from 18 Brazilian states, providing inter and multidisciplinary services, including medical and dental treatment, genetic counseling, and psychological and social assistance.^26^ The comparison group of children without NSCL±P was recruited in the pediatric dentistry clinic at the Federal University of Paraná (Curitiba, Paraná, Brazil). Children with syndromes or craniofacial anomalies other than oral clefts and dental anomalies were excluded.

NSCL±P is a group of clefts that includes cases of isolated cleft lip (CL), cleft lip and alveolus (CLA), and cleft lip and palate (CLP). Then, children presenting any of these types of clefts were included. Isolated cleft palate (CP) is considered a distinct congenital malformation, presenting different embryologic origins to NSCL±P.^27^ Thus, CP cases were not included in the current study. The determination of the cleft type was based on clinical examination and confirmed by medical records. Data collection was performed from January 2022 until August 2023.

The sample size was calculated considering the association data between clefts and tooth abnormalities.^4^ The proportion of tooth abnormalities among individuals with clefts was set at 38.8%, with an 80% power determination, a 95% confidence interval (95%CI), and a 3.14 odds ratio (OR). To cover possible losses, the sample was increased by 20%. Thus, the final sample size was estimated to be between 100 and 120 children per group.

### TA definition

Patients presenting one or more panoramic radiographs were included in this evaluation. One examiner (G.F.S), a pediatric dentist, was trained to diagnose TA in panoramic radiographs in both groups. Permanent teeth TA (excluding third molars) was assessed using panoramic radiographs obtained from patient’s records and was defined based on the age of patients, and when initial tooth formation should be visible in the radiographs, according to dental chronology.^28^ In the NSCL±P group, only TA located outside the cleft area was considered. Thus, in cases of unilateral or bilateral CL, any permanent tooth agenesis was considered. For unilateral right CLA and CLP, all permanent teeth, except the right maxillary lateral incisor, were considered, and for unilateral left CLA and CLP, all except the left maxillary lateral incisor. In bilateral CLA and CLP cases, both maxillary lateral incisors were not considered. In the comparison group, the agenesis of any permanent teeth was contemplated.

All evaluated radiographs presented high-quality requirements and were examined digitally in a darkroom by a single trained examiner. When more than one panoramic radiograph was available for a child, the extra one was assessed to confirm the diagnosis. Intra-examiner reliability was assessed by repeating the evaluation of ten randomly selected panoramic radiographs at a one-month interval. The Kappa coefficient (kappa=1) indicated perfect agreement between both evaluation times.

### DNA extraction and genotyping analysis

Buccal cells were obtained using a 5 ml mouthwash of saline solution. Genomic DNA was extracted from buccal cells following the protocol proposed by Küchler, et al.^29^ (2012). The concentration and purity of the DNA were determined by spectrophotometry (Nanodrop 2000, Thermo Scientific, Wilmington, DE, USA).

We selected genetic polymorphisms in *TGFB1* (rs4803455 and rs1800470), *TGFBR2* (rs764522 and rs3087465), *EGF* (rs4444903 and rs2237051), and *EGFR* (rs2227983), which are genes suggested to be involved in the craniofacial development.^10,13,14,17,18,21,22,30-33^ Genetic polymorphisms were selected based on their global minor allele frequency (>10%) and their possible impact on palatogenesis and odontogenesis. Thus, we hypothesized that these genes play a role in odontogenesis and that common polymorphisms in these genes could be involved in TA. Gene characteristics and selected polymorphisms are presented in Table 1.

**Table 1:** Characteristics of the selected genes for this study.

Gene name and acronym	Chromosome	Gene function in craniofacial development	Genetic Polymorphism	Base change	Function	Global MAF
Transforming Growth Factor Beta 1 (*TGFB1*)	19q13.2	Acts in cell proliferation, growth, and fusion of the palatal shelves.^14^^,^^18^	rs4803455	C/A	Intron	0.480
		Regulates the connective tissue growth factor expression, which plays a role in the formation of secondary palate.^21^	rs1800470	A/G	Missense	0.495
		Regulates odontoblastic differentiation.^30^				
		Regulates tooth root formation.^33^				
Transforming Growth Factor Beta Receptor 2 (*TGFBR2*)	3p24.1	Acts in the fusion of the palatal shelves during palate development.^31^	rs764522	C/G	Upstream	0.253
		Regulates odontoblasts cells differentiation.^32^	rs3087465	A/G	Upstream	0.248
Epidermal Growth Factor (*EGF*)	4q25	Acts in the degeneration of the medial edge epithelial cells during palate formation.^13^^,^^17^	rs4444903	A/G	Non-Coding Transcript	0.424
		It is expressed in the developing mandible before the appearance of dental lamina, having a function in tooth development.^16^	rs2237051	A/G	Missense	0.412
		Inhibits the morphogenesis of teeth and prevents odontoblast and ameloblasts cell differentiation.^22^				
Epidermal Growth Factor Receptor (*EGFR*)	7p11.2	Participates in craniofacial development and palate closure.^17^	rs2227983	A/G	Missense	0.264
		It is expressed in the dental epithelium and the dental mesenchyme during the initial stages of odontogenesis.^22^				

Note: Obtained from databases: http://www.thermofisher.com and.

Allelic discrimination assays were analyzed using real-time polymerase chain reaction (PCR) for genotyping using the StepOnePlus real-time PCR system (Thermo Fisher Scientific, Waltham, Mass., USA). Genotype success rate ranged from 97.2% to 99.5%.

### Statistical analysis

The association between NSCL±P and TA was analyzed using Chi-square. Poisson regression with robust variance was carried out to evaluate the relationship of the dependent variables NSCL±P and TA, with the independent variables “sex” and “genes encoding growth factors phenotypes.” NSCL±P and TA were considered dependent variables and were categorized according to their presence as “yes” or “no”. Genotypes were analyzed as additive, dominant, and recessive models. Multiple models using the stepwise approach were performed for independent variables with p<0.20 in the univariable model. A 5% significance level was adopted (p<0.05). All the analyses were performed using the Statistical Package for Social Sciences 20.0 software (SPSS, Chicago, IL, USA).

The Hardy-Weinberg Equilibrium (HWE) was assessed for each genetic polymorphism using the Chi-square test (wpcalc.com/en/equilibrium-hardy-weinberg) for the total sample. Deviations from HWE were considered when Chi-square > 3.84.

The potential functional impact of the polymorphism associated with the dependent variables was predicted using PolyPhen-2,^34^ a tool that estimates the likelihood of a variant affecting protein function based on sequence and structural data.

## Results

**Figure 1:**
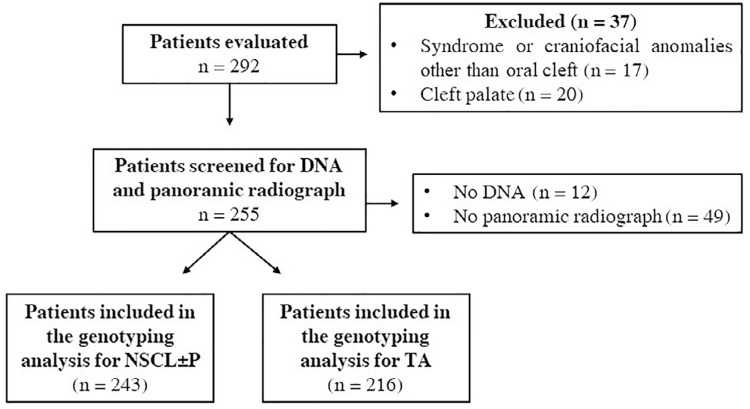
Flow diagram of the study

This study is part of a larger project whose aim was to investigate the relation between NSCL±P and tooth abnormalities. A total of 292 children were evaluated. Of those, thirty-seven (n=37) were excluded for the following reasons: the presence of syndromes or craniofacial anomalies other than cleft (n=17) and the presence of CP (n=20). Thus, 255(n=255) patients were screened for DNA samples and panoramic radiographs. Of them, 243 presented DNA and were included in the genotyping analysis for NSCL±P, 127 patients with NSCL±P and 116 without NSCL±P, and 216 presented both DNA and a panoramic radiograph and were included in the genotyping analysis for TA (Figure 1). The characteristics of the study population are presented in Table 2.

**Table 2:** Characteristics of the study population in relation to the groups (Curitiba, Paraná, Brazil, n=243, 2024)*.

Variable	Categories		Group n (%)		p-value
			NSCL±P (n=127)	Control (n=116)	
Type of cleft	CL		42 (33.1)	-	-
	CLP		85 (66.9)	-	-
Sex	Female		52 (40.9)	64 (55.2)	**0.027**
	Male		75 (59.1)	52 (44.8)	
Age (years)	Mean (SD)		8.80 (2.14)	8.58 (2.03)	0.385**
TA	One or more teeth	Yes	25 (23.8)	7 (6.2)	**<0.01**
		No	80 (76.2)	105 (93.8)	
	Maxillary lateral incisor	Yes	5 (4.8)	3 (2.7)	0.326*
		No	100 (95.2)	109 (97.3)	
	Maxillary second premolar	Yes	17 (16.2)	0 (0.0)	<0.01*
		No	88 (83.8)	112 (100)	
	Mandibular second premolar	Yes	5 (4.8)	4 (3.6)	0.460*
		No	100 (95.2)	108 (96.4)	

Abbreviations: NSCL±P, non-syndromic cleft lip with or without cleft palate; CL, isolated cleft lip; CLA, cleft lip and alveolus; TA, tooth agenesis; SD, standard deviation.

TA: a total of 216 patients presented a panoramic radiograph and were evaluated for TA.

p-value: Chi-square test, *Fisher’s exact test.

**Mann-Whitney U test.

Bold means statistically significant difference.

TA was significantly more frequent in the NSCL±P group (23.8%) than in comparison group (6.2%) (p<0.01). In the sample, the teeth affected by TA were the maxillary lateral incisors (only the ones located outside the cleft area in the NSCL±P group were considered), and the maxillary and mandibular second premolars. The absence of maxillary second premolars was only observed in the group with NSCL±P (n=17). Two patients in the NSCL±P group presented more than one TA (Table 2).

Considering the association between NSCL±P and the independent variables, a significant association was observed between cleft presence and sex (PR_c_= 1.35; p=0.016), with male individuals presenting a higher prevalence of NSCL±P (60.2%) than female (44.6%) in the univariate analysis (Table 3). In the multivariable analysis, the rs2237051 in *EGF* was significantly associated with NSCL±P independent of the other variables (PR_a_=1.41; p=0.042). Individuals presenting at least one allele A (AA/AG) had a lower prevalence of NSCL±P than those presenting genotype GG (Table 3). The bioinformatic prediction analysis for rs2237051 indicated that this polymorphism is unlikely to affect protein function, as the predicted score was consistent with a benign impact.

**Table 3:** Crude prevalence ratio of NSCL±P according to the characteristics of the sample and the growth factors encoding genes genotype (Curitiba, Paraná, Brazil, n=217, 2024).

Variable	Categories	NSCL±P		PRc (CI 95%)	p-value	PRa (CI 95%)*
		Yes n (%)	No n (%)			
Sex	Female	54 (44.6)	67 (55.4)	Reference	**0.016**	Reference
	Male	80 (60.2)	53 (39.8)	1.35 (1.06-1.72)		1.23 (0.96-1.57)
**rs4803455 (*TGFB1*)**						
Additive model	CC	44 (50.0)	44 (50.0)	0.93 (0.71-1.21)	0.583	-
	AA	18 (56.2)	14 (43.8)	1.04 (0.74-1.48)	0.812	-
	CA	62 (53.9)	53 (46.1)	Reference		-
Dominant model	CC	44 (50.0)	44 (50.0)	0.91 (0.71-1.19)	0.516	-
	CA + AA	80 (54.4)	67 (45.6)	Reference		-
Recessive model	AA	18 (56.2)	14 (43.8)	1,07 (0.77-1.50)	0.661	-
	CA + CC	106 (52.2)	97 (47.8)	Reference		-
**rs1800470 (*TGFB1*)**						
Additive model	AA	31 (59.6)	21 (40.4)	1.13 (0.85-1.49)	0.397	-
	GG	29 (46.8)	33 (53.2)	0.88 (0.64-1.21)	0.446	-
	AG	65 (52.8)	58 (47.2)	Reference		-
Dominant model	GG	29 (46.8)	33 (53.2)	0.85 (0.63-1.15)	0.294	-
	AG + AA	96 (54.9)	79 (45.1)	Reference		-
Recessive model	AA	31 (59.6)	21 (40.4)	1.17 (0.90-1.53)	0.237	-
	AG + GG	95 (50.8)	91 (49.2)	Reference		-
**rs764522 (*TGFBR2*)**						
Additive model	CC	77 (52.4)	70 (47.6)	Reference		-
	GG	5 (45.5)	6 (54.5)	0.87 (0.45-1.69)	0.676	-
	GG	44 (55.7)	35 (44.3)	1.06 (0.83-1.36)	0.630	-
Dominant model	CC	77 (52.4)	70 (47.6)	Reference	0.756	-
	CG + GG	49 (54.4)	41 (45.6)	1.04 (0.81-1.33)		-
Recessive model	GG	5 (45.5)	6 (54.5)	0.85 (0.44-1.64)	0.626	-
	CG + CC	121 (53.5)	105 (46.5)	Reference		-
**rs3087465 (*TGFBR2*)**						
Additive model	AA	8 (33.3)	16 (66.7)	0.59 (0.33-1.07)	0.082	-
	GG	63 (56.2)	49 (43.8)	Reference		-
	AG	54 (54.0)	46 (46.0)	0.96 (0.75-1.22)	0.743	-
Dominant model	GG	63 (56.2)	49 (43.8)	1.12 (0.88-1.43)	0.336	-
	AG + AA	62 (50.0)	62 (50.0)	Reference		-
Recessive model	AA	8 (33.3)	16 (66.7)	0.60 (0.34-1.08)	0.088	0.64 (0.36-1.13)
	AG + GG	117 (55.2)	95 (44.8)	Reference		Reference
**rs4444903 (*EGF*)**						
Additive model	AA	36 (59.0)	25 (41.0)	1.17 (0.89-1.54)	0.259	-
	GG	28 (48.3)	30 (51.7)	0.96 (0.70-1.32)	0.791	-
	AG	61 (50.4)	60 (49.6)	Reference		-
Dominant model	AA	36 (59.0)	25 (41.0)	1.19 (0.92-1.53)	0.189	0.93 (0.65-1.31)
	AG + GG	89 (49.7)	90 (50.3)	Reference		Reference
Recessive model	GG	28 (48.3)	30 (51.7)	0.91 (0.67-1.22)	0.517	-
	AG + AA	97 (53.3)	85 (46.7)	Reference		-
**Variable**	**Categories**	**NSCL±P**		**PRc (CI 95%)**	**p-value**	PRa (CI 95%)*
		**Yes n (%)**	**No n (%)**			
**rs2237051 (*EGF*)**						
Additive model	AA	26 (49.1)	27 (50.9)	1.01 (0.72-1.40)	0.971	-
	GG	42 (61.8)	26 (38.2)	1.27 (0.97-1.64)	0.076	-
	AG	59 (48.8)	62 (51.2)	Reference		-
Dominant model	GG	42 (61.8)	26 (38.2)	1.26 (0.99-1.61)	0.056	1.41 (1.01-1.95)
	AG + AA	85 (48.9)	89 (51.1)	Reference		Reference
Recessive model	AA	26 (49.1)	27 (50.9)	0.92 (0.68-1.24)	0.582	-
	AG + GG	101 (53.4)	88 (46.6)	Reference		-
**rs2227983 (*EGFR*)**						
Additive model	AA	10 (55.6)	8 (44.4)	1.06 (0.68-1.65)	0.801	-
	GG	74 (52.5)	67 (47.5)	Reference		-
	AG	42 (52.5)	38 (47.5)	1.00 (0.77-1.30)	0.998	-
Dominant model	GG	74 (52.5)	67 (47.5)	Reference	0.930	-
	AG + AA	52 (53.1)	46 (46.9)	1.01 (0.79-1.29)		-
Recessive model	AA	10 (55.6)	8 (44.4)	1.06 (0.69-1.63)	0.797	-
	AG + GG	116 (52.5)	105 (47.5)	Reference		-

Bold means statistically significant difference.

Abbreviations NSCL±P , non-syndromic cleft lip and/or palate; PRc, crude prevalence rate; CI 95%, confidence interval of 95%; PRa, adjusted prevalence rate.

*To perform the multiple Poisson regression analysis, independent variables with p<0.20 in the univariable model were added to the multiple models. The PRa was adjusted by the following independent variables: sex, dominant model for rs4444903 (*EGF*), and dominant model for rs2237051 (*EGF*).

Regarding TA data, only cleft presence was associated with a higher prevalence of TA regardless of other variables (PR_a_=3.70; p=0.001). There was no association between TA and the investigated genetic polymorphisms (Table 4). Only the rs1800470 in *TGFB1* showed a borderline association with TA presence when the cleft presence was also considered (p=0.065).

**Table 4:** Crude prevalence ratio of TA according to the characteristics of the sample and the growth factors encoding gene genotype (Curitiba, Paraná, Brazil, n=217, 2024).

Variable	Categories	TA		PRc (CI 95%)	p-value	PRa (CI 95%)*
		Yes n (%)	No n (%)			
NSCL±P	Yes	25 (23.8)	80 (76.2)	3.81 (1.72–8.43)	**0.001**	3.70 (1.67–8.20)
	No	7 (6.2)	105 (93.8)	Reference		Reference
Sex	Female	17 (16.7)	85 (83.3)	1.29 (0.67-2.43)	0.454	-
	Male	15 (13.0)	100 (87.0)	Reference		-
**rs4803455 (*TGFB1*)**						
Additive model	CC	14 (18.9)	60 (81.1)	1.35 (0.69-2.66)	0.383	-
	AA	2 (7.7)	24 (92.3)	0.55 (0.13-2.27)	0.408	-
	CA	14 (14.0)	86 (86.0)	Reference	0.383	-
Dominant model	CC	14 (18.6)	60 (81.1)	1.49 (0.77-2.87)	0.235	-
	CA + AA	16 (12.7)	110 (87.3)	Reference		-
Recessive model	AA	2 (7.7)	24 (92.3)	0.48 (0.12-1.89)	0.292	-
	CA + CC	28 (16.1)	146 (83.9)	Reference		-
**rs1800470 (*TGFB1*)**						
Additive model	AA	5 (12.2)	36 (87.8)	0.95 (0.36-2.47)	0.915	-
	GG	11 (21.2)	41 (78.8)	1.65 (0.80-3.37)	0.173	-
	AG	14 (12.8)	95 (87.2)	Reference		
Dominant model	GG	11 (21.2)	41 (78.8)	1.67 (0.85-3.27)	0.135	1.83 (0.96-3.50)
	AG + AA	19 (12.7)	131 (87.3)	Reference		Reference
Recessive model	AA	5 (12.2)	36 (87.8)	0.78 (0.32-1.93)	0.598	-
	AG + GG	25 (15.5)	136 (84.5)	Reference		-
**rs764522 (*TGFBR2*)**						
Additive model	CC	18 (15.0)	102 (85.0)	Reference		-
	GG	0 (0.0)	11 (100.0)	-	-	-
	CG	11 (15.5)	60 (85.0)	1.03 (0.52-2.06)	0.927	-
Dominant model	CC	18 (15.0)	102 (85.0)	Reference	0.753	-
	CG + GG	11 (13.4)	71 (86.6)	0.89 (0.45-1.79)		-
Recessive model	GG	0 (0.0)	11 (100.0)	-	-	-
	CG + CC	29 (15.2)	162 (84.8)	Reference		-
**rs3087465 (*TGFBR2*)**						
Additive model	AA	1 (4.5)	21 (95.5)	0.26 (0.04-1.87)	0.181	-
	GG	16 (17.4)	76 (82.6)	Reference		-
	AG	12 (14.0)	74 (86.0)	0.80 (0.40-1.60)	0.531	-
Dominant model	GG	16 (17.4)	76 (82.6)	1.44 (0.73-2.84)	0.287	-
	AG + AA	13 (12.0)	95 (88.0)	Reference		-
Recessive model	AA	1 (4.5)	21 (95.5)	0.29 (0.04-2.02)	0.211	-
	AG + GG	28 (17.7)	150 (84.3)	Reference		-
**rs4444903 (*EGF*)**						
Additive model	AA	6 (11.8)	45 (88.2)	0.73 (0.30-1.73)	0.471	-
	GG	7 (14.6)	41 (85.4)	0.90 (0.40-2.03)	0.801	-
	AG	17 (16.2)	88 (83.8)	Reference	0.471	-
Dominant model	AA	6 (11.8)	45 (88.2)	0.75 (0.32-1.73)	0.500	-
	AG + GG	24 (15.7)	129 (84.3)	Reference		-
Recessive model	GG	7 (14.6)	41 (85.4)	0.99 (0.45-2.16)	0.978	-
	AG + AA	23 (14.7)	133 (85.3)	Reference		-
**rs2237051 (*EGF*)**						
Additive model	AA	6 (13.0)	40 (87.0)	0.77 (0.33-1.84)	0.563	-
	GG	7 (12.1)	51 (87.9)	0.72 (0.32-1.63)	0.426	-
	AG	17 (16.8)	84 (83.2)	Reference	0.563	-
Dominant model	GG	7 (12.1)	51 (87.9)	0.77 (0.35-1.70)	0.519	-
	AG + AA	23 (15.6)	124 (84.4)	Reference		-
Recessive model	AA	6 (13.0)	40 (87.0)	0.86 (0.38-1.99)	0.731	-
	AG + GG	24 (15.1)	135 (84.9)	Reference		-
**rs2227983 (*EGFR*)**						
Additive model	AA	1 (5.9)	16 (94.1)	0.41 (0.06-2.92)	0.377	-
	GG	17 (14.2)	103 (85.8)	Reference		-
	AG	12 (18.2)	54 (81.8)	1.28 (0.65-2.52)	0.469	-
Dominant model	GG	17 (14.2)	103 (85.8)	Reference	0.768	-
	AG + AA	13 (15.7)	70 (84.3)	1.10 (0.57-2.15)		-
Recessive model	AA	1 (5.9)	16 (94.1)	0.38 (0.05-2.60)	0.322	-
	AG + GG	29 (15.6)	157 (84.4)	Reference		-

Abbreviations: TA, tooth agenesis; NSCL±P , non-syndromic cleft lip and/or palate; PRc, crude prevalence rate; CI 95%, confidence interval of 95%; PRa, adjusted prevalence rate.

*To perform the multiple Poisson regression analysis, independent variables with p<0.20 in the univariable model were added to the multiple models. The PRa was adjusted by the following independent variables: NSCL±P and dominant model for rs1800470 (*TGFB1*).

## Discussion

Craniofacial development and odontogenesis are complex and interrelated processes coordinated and regulated by several genetic factors.^11^ Alterations in these processes may result in abnormalities in orofacial and dental structures, like NSCL±P and TA.^35^ In this study, individuals with NSCL±P presented 270% more prevalence of TA outside of cleft area, corroborating previous studies in different populations.^7,8,10,11,36^ In the Netherlands, Bartzela, et al.^8^ (2023) observed that TA outside the cleft area was present in 20.9% of patients with unilateral NSCL±P. Mangione, et al.^7^ (2018) found that 54.7% of French patients with oral clefts presented TA outside the cleft area. In the Brazilian population, the reported prevalence of TA outside the cleft area ranges from 28.6% to 47.5%.^10,11,36^

Only cases of TA occurring outside the cleft area were considered here. We did not consider agenesis of lateral incisors occurring inside the cleft area. In humans, the lateral incisor is often affected by developmental anomalies. The medial nasal and maxillary processes, which form the upper lip and alveolar ridge, provide tissue for lateral incisors formation, therefore, failures in the fusion of these processes may be a local factor associated with lateral incisor agenesis.^1^ TA located outside the cleft area may suggest that this dental abnormality shares a similar genetic background with orofacial clefts.^5^

In this study, individuals with GG in rs2237051 (*EGF*) presented a statistically higher prevalence of NSCL±P than individuals with AA/AG. This polymorphism is a missense variant, which is a change in the DNA sequence that leads to the production of a different amino acid in the translated protein. In the case of a conservative substitution, one amino acid is replaced by another with similar chemical properties, generally not affecting the function of the protein. In rs2237051, methionine is replaced by isoleucine at amino acid position 708 of the protein, characterizing a conservative substitution. A bioinformatic prediction analysis for rs2237051 using PolyPhen-2^34^ further indicated that this polymorphism is unlikely to alter protein function. A conservative missense mutation, while typically less disruptive, may still have an impact on protein function. Even though it does not directly alter the structure of the protein, it can influence the biochemical properties of the protein, like reaction kinetics or stability, without completely abolishing its function.^37^ It is pointed out in the literature that *EGF* is required for epithelial cell growth and differentiation, including the degeneration of the medial edge epithelial cells during palate formation.^13,17^ Thus, it is possible to suggest that alterations in this gene may affect adequate palate development, resulting in the occurrence of NSCL±P. According to our knowledge, this is the first study to investigate the role of this polymorphism in craniofacial developmental alterations. Therefore, future studies should investigate the association between these polymorphisms and the occurrence of NSCL±P in different populations.

No association was found between NSCL±P and the rs444903, which is consistent with previous findings of a study also conducted in the Brazilian population.^38^ On the other hand, a study in a Chinese population^39^ found that individuals with the GA genotype in rs444903 had a reduced risk of NSCL±P. Expressivity of genetic variants is influenced by several factors, including environmental influences, which can lead the same gene to produce a wide variety of phenotypes in different individuals and populations.^40^ This variability could be an explanation for the contrasting findings observed across these studies.

TA was not associated with the evaluated polymorphisms. Only rs1800470 in *TGFB1* showed a borderline association with TA presence when the cleft presence was also considered. During odontogenesis, *TGFB1* is reported to regulate odontoblastic differentiation,^30^ and root formation.^33^ Rs1800470 is a non-conservative missense mutation in which the amino acid cytosine is replaced by thymine. In studies with humans, this polymorphism was associated with decreased tooth size.^23^ It is hypothesized that TA and decreased tooth size share a similar genetic background,^41^ which could explain these findings and reinforce the possible role of this polymorphism in odontogenesis. However, this borderline association should be interpreted with caution, since this study presented a limited sample size and further instigation with a larger sample size is necessary to confirm the possible role of this polymorphism in TA occurrence.

Studies with animals suggest a participation of *TGBR2* and *EGFR* in the craniofacial development, expressed during palatogenesis^17,31,42^ and in the beginning of odontogenesis.^22,43^ In humans, *TGFBR2* is associated with Loeys–Dietz syndrome, an alteration characterized by the hypertelorism triad, cleft palate or bifid uvula, arterial aneurysms and arterial tortuosity.^44^ Besides that, the rs3087465 in this gene was associated with mandibular retrognathism.^24^ The evidence in humans about *EGFR*, however, is scarce and presents contradictory results.^45^ In this study none of the polymorphism evaluated in *TGFB2* and *EGFR* was associated with NSCL±P or TA. Further studies are needed to confirm the possible function of these genes in craniofacial malformations.

A previous systematic review^5^ demonstrated that genes contributing to craniofacial development are involved in the co-occurrence of clefts and TA in humans, including the msh homeobox 1 (*MSX1*), the paired box 9 (*PAX9*), and the interferon regulatory transcription factor 6 (*IRF6*). However, in this study, due to the lower occurrence of TA observed in the group without NSCL±P, it was not possible to evaluate the co-occurrence of these craniofacial abnormalities in the genetic analysis, which may be considered a limitation.

Studies with patients with NSCL±P have many methodological challenges, since participants are mainly recruited from specific centers, resulting in convenience samples with limited size, as observed in the present study. Considering the unpredictability in estimating the frequency of genetic polymorphisms within the sample, especially given the number of polymorphisms analyzed, the Hardy-Weinberg equilibrium was calculated to account for potential frequency imbalances. However, the power of comparisons between the polymorphism and NSCL±P and TA varied significantly. On the other hand, the primary aim of the study was to evaluate the association between NSCL±P and TA, for which the statistical power was very high. It is important to highlight that this study was conducted in a specific population, which may restrict the generalizability of the findings to other groups. Moreover, it highlights the need for further well-designed research to explore genetic factors linked to dental phenotypes in individuals with NSCL±P.

Our findings contribute to a more comprehensive understanding about genetic factors and phenotypes associated with craniofacial alterations. Considering that the type of cleft and the local of occurrence of TA may impact the results, we excluded individuals with CP due to its different embryological origin and we did not consider TA inside the cleft area, avoiding biased findings. Additionally, the control group of children without NSCL±P was recruited from a similar population, ensuring more accurate comparisons. Notably, the high prevalence of TA in individuals with NSCL±P highlights the importance of early dental evaluations and interventions for these patients. Furthermore, understanding the genetic factors contributing to these conditions may facilitate more personalized treatment strategies, enabling the identification of patients at greater risk for dental anomalies and enhancing the care and management of individuals with craniofacial abnormalities.

## Conclusion

Considering the findings in these populations, it was concluded that NSCL±P is significantly associated to TA outside of the cleft area and with the rs2237051 in *EGF*, and that polymorphisms in *TGFB1, TGFBR2, EGF* and *EGF2* are not associated with TA.
